# Effect of Host Genotype on Symbiont Titer in the Aphid—*Buchnera* Symbiosis

**DOI:** 10.3390/insects2030423

**Published:** 2011-09-16

**Authors:** Kevin J. Vogel, Nancy A. Moran

**Affiliations:** 1 Department of Ecology and Evolutionary Biology, Biological Sciences West, Room 301, 1041 E. Lowell St., The University of Arizona, Tucson, AZ 85721-0088, USA; 2 EEB West Campus, Yale University, PO Box 27388, West Haven, CT 06516-7388, USA; E-Mail: nancy.moran@yale.edu

**Keywords:** symbiont titer, *Buchnera aphidicola*, aphid, nutritional symbiosis

## Abstract

Obligate nutritional symbioses require balance between the energetic needs of the host and the symbiont. The resident symbiont population size within a host may have major impacts on host fitness, as both host and symbiont consume and supply metabolites in a shared metabolite pool. Given the massive genome degradation that is a hallmark of bacterial endosymbionts of insects, it is unclear at what level these populations are regulated, and how regulation varies among hosts within natural populations. We measured the titer of the endosymbiont *Buchnera aphidicola* from different clones of the pea aphid, *Acyrthosiphon pisum*, and found significant variation in titer, measured as *Buchnera* genomes per aphid genome, among aphid clones. Additionally, we found that titer can change with the age of the host, and that the number of bacteriocytes within an aphid is one factor likely controlling *Buchnera* titer. *Buchnera* titer measurements in clones from a sexual cross indicate that the symbiont genotype is not responsible for variation in titer and that this phenotype is likely non-heritable across sexual reproduction. Symbiont titer is more variable among lab-produced F_1_ aphid clones than among field-collected ones, suggesting that intermediate titer is favored in natural populations. Potentially, a low heritability of titer during the sexual phase may generate clones with extreme and maladaptive titers each season.

## 1. Introduction

Bacterial endosymbionts are common associates of insects, often providing essential nutrients that are absent from the host diet [1]. *Buchnera aphidicola*, the primary symbiont of aphids, has become a model system for the study of these relationships. This member of the Gammaproteobacteria produces essential amino acids that are rare in the diet of aphids, phloem sap [2,3]. Early investigations of these symbionts suggested that the host controlled the titer of the symbionts [4], and later genomic studies revealed that reductive genome evolution in these symbionts resulted in the loss of many genes necessary for regulation of cell processes including division and growth [3,5]. These observations suggest that the symbiont is unable to control its own replication, and that the host plays a major role in regulating the titer of symbionts.

Despite the loss of regulatory mechanisms to control symbiont division and metabolism, variation in symbiont genotypes may contribute to differences in symbiont titer. *Buchnera*'s high mutation rate [6], asexuality, and small population size of the symbiont [7,8] can lead to disruptions of processes that are likely essential to both symbiont and host [9]. *Buchnera* titer may also have been shaped by selection on the host, as the symbiont has little or no ability to alter gene expression in response to differences in amino acid content of phloem [10], which is known to vary among and between host plant species [11]. Symbiont titer may therefore be a mechanism for regulating the amino acid metabolism of the system.

*A. pisum* is a cyclical parthenogen, like many aphids, and undergoes several generations of clonal reproduction followed by a single generation of sexual reproduction [12]. In such life cycles, traits with epistatic or dominance genetic variance are heritable during clonal reproduction but not across the sexual reproductive phase [13,14]. Symbiont titer may be one such phenotype.

To assess variation in symbiont titer, we measured the titer of *Buchnera* in populations of the pea aphid, *Acyrthosiphon pisum*. To examine the underlying basis of the variation, we measured the number of bacteriocytes, the specialized cells in which *Buchnera* resides, between the clones, as well as the number of *Buchnera* within a bacteriocyte and the relationship of titer to amino acid requirements of the clones. Two clones with high and low titer were bred to produce F_1_ offspring, which were then screened for titer and bacteriocyte number.

## 2. Experimental Section

### 2.1. Aphid Clones

Parthenogenetic *A. pisum* females were collected from across the United States between 1998 and 2007 (Table S1). For each clone, a single female was used to establish clonal lineages maintained continuously under long day (16:8 L:D) conditions. Experimental aphid lines were kept in a growth chamber at 20 °C on *Vicia faba* seedlings in cup cages [15]. Short day conditions were used to induce sexual forms for clones 8–10–1 and 5A, which were reciprocally mated to yield F_1_ aphid clones, as described by Moran and Dunbar [16]. Individuals hatching from the sexually produced eggs were isolated and allowed to establish full sib clonal lineages under long day conditions. For all experiments, clones were divided into 3 sub-clones and allowed to reproduce for 3 generations prior to collection to control for maternal and environmental effects. Each experiment was replicated twice, beginning with the establishment of new subclones.

### 2.2. Bacteriocyte Counts

Adult viviparous females were placed on fresh *V. faba* seedlings and allowed to deposit nymphs for 12 hours, after which the adults were removed and the nymphs allowed to develop for 6 days, to their 4^th^ instar. Fourth instar aphids were dissected in buffer A (250 mM sucrose, 35 mM Tris-HCl, 25 mM KCl, 10 mM MgCl_2_) in a watch glass. All bacteriocytes were identified, separated and counted under 6× magnification.

### 2.3. DNA Extractions

Adult viviparous *A. pisum* were placed on fresh *V. faba* seedlings and allowed to deposit nymphs for 12 hours. Nymphs were either allowed to develop for six days to their fourth instar or immediately collected at their first instar. Individual nymphs were collected in pestle tubes, frozen in liquid nitrogen, and crushed with a pestle. The resulting homogenized tissue was treated according to the Qiagen DNEasy kit.

To isolate DNA from individual bacteriocytes, single bacteriocytes from the aphids used for bacteriocyte counts were collected in pestle tubes, frozen in liquid nitrogen and crushed. Due to the small amount of starting material, the resulting homogenate was treated with lysis buffer [17] and then washed twice with phenol:chloroform:isoamyl alcohol 25:24:1, then once with chloroform. The DNA was then precipitated with sodium acetate and ethanol and resuspended in low TE (10 mM EDTA, 100 mM Tris-HCl). All DNA was treated with RNAse I at 37 °C for 30 minutes. Three aphids from each of the 3 subclones from each clone were used for the experiments.

### 2.4. Quantitative PCR

*Buchnera* titer was measured by comparing the number of *Buchnera* genomes to the number of aphid genomes using a single copy gene from both the aphid and the symbiont. This provided a rough correction for size differences between aphid clones, though some aphid cells are polyploid [18]. Aphid genomes were counted by assessing copy number of the gene encoding elongation factor 1-alpha (*ef1α*), while *Buchnera* genomes were counted by using the gene encoding adenosylmethionine-8-amino-7-oxononanoate aminotransferase (*bioA*). Primers used were ApEF1-alpha 107F 5′ - CTGATTGTGCCGTGCTTATTG - 3′, ApEF1-alpha 246R 5′ - TATGGTGGTTCAGTAGAGTCC - 3′, BuchAPS *bioA* 374F 5′ - AGTATTGGCAAGCATTAGGGC - 3′, BuchAPS *bioA* 526R 5′ - AAAAGAAGAAACTGGTCGTC - 3′. Standards of 10^7^ copies were prepared according to the method of [10] for each gene. For each sample from 1^st^ instar aphids, the number of copies of *ef1α* and *bioA* were compared on a Roche LightCycler using the FastStart DNA Master^plus^ SYBR Green I kit according to the kit instructions. Copy number was determined using LightCycler 3.0 software in comparison to the standards for each gene. For individual bacteriocytes, only the copy number of *bioA* was assessed, though the bacteriocytes are polyploid [18]. For each clone, three aphids from each subclone were tested, and each clone was measured twice, starting with the initiation of new subclones.

### 2.5. Statistical Analysis

*Buchnera* titer was determined by the ratio of *Buchnera* genomes to *A. pisum* genomes. The regression of *Buchnera* genome copies and aphid genome copies was linear but the slopes of the variables were unequal and the intercepts were non-zero, so ANCOVA analysis for unequal slopes was applied in JMP 8 (SAS). *Buchnera* genome copy number of individual bacteriocytes was log transformed and analyzed by ANOVA. Bacteriocyte counts were normally distributed and analyzed by MANOVA. Summary statistics are included in Table S2.

## 3. Results and Discussion

### 3.1. *Buchnera* Titer

**Figure 1:**
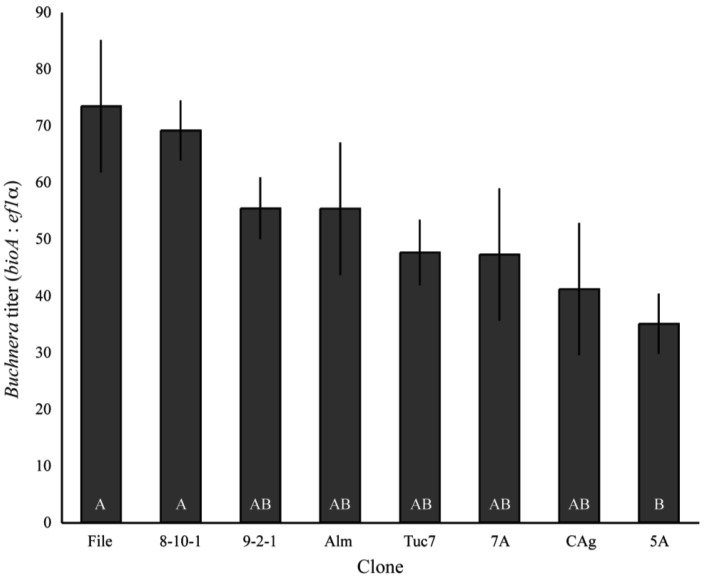
Average *Buchnera* titer of 1^st^ instar aphids from lab-reared *A. pisum* clones. Titer is the ratio of a single-copy *Buchnera* gene (*bioA*) to a single copy *A. pisum* gene (*ef1α*). Clones exhibited significant differences in titer (F_9,54_ = 3.21, p = 0.0019, ANCOVA). Bars with different letters are significantly different (p < 0.05, Tukey's HSD). Error bars are ± SE.

*Buchnera* titer (*Buchnera* genomes per aphid genome) varied significantly among field-collected clones of *A. pisum* in the first instar (F_9,95_ = 3.21, p = 0.0019, ANCOVA), with the lowest titer clone–5A–having an average of 35 *Buchnera* genomes per aphid genome, while the highest titer clone–File–had an average titer of 73.4 (Figure 1). The copy number of both *ef1α* and *bioA* varied significantly between clones (p < 0.0001 ANOVA), but the variation in *ef1α* copy number only explained 4.4% of the variance in *Buchnera* titer while *bioA* copy number explained 36.6% of the variance based on measuring effects of each variable independently.

**Figure 2:**
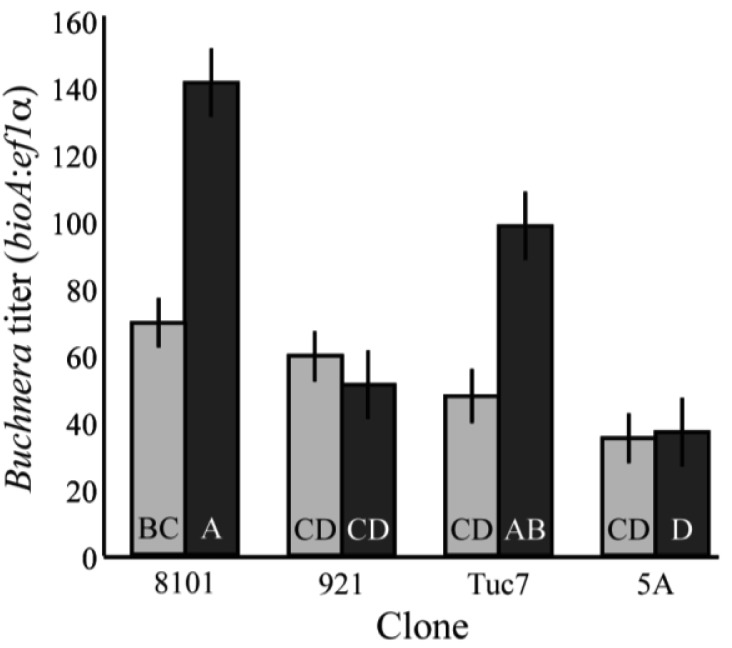
Titer of 4^th^ instar *A. pisum* in comparison to 1^st^ instar aphids from the same clones. Light bars–1^st^ instar aphids; dark bars 4^th^ instar aphids. There was a significant difference in titer between 1^st^ and 4^th^ instar clones (F_7,97_ = 15.37, p < 0.0001; ANCOVA). Bars with different letters are significantly different (p < 0.05, Tukey's HSD). Error bars are ± SE.

*Buchnera* titer also varies across the development of the host. Four clones were tested, and there were significant changes in the titer of *Buchnera* from 1^st^ to 4^th^ instar (F_7,97_ = 10.37, p < 0.0001; ANCOVA, Figure 2), with clones 8-10-1 and Tuc7 increasing significantly in titer (p = 0.0005 and p = 0.027, respectively student's t-test), and the titer in clones 5A and 9-2-1 not changing significantly (p = 0.82, p = 0.33, respectively student's t-test). Titer varied significantly between the clones at the 4^th^ instar (Figure 2), with clones 8-10-1 and Tuc7 exhibiting similar titer and clones 5A and 9-2-1 having significantly lower titer (p < 0.05, Tukey's HSD). The copy number of both *Buchnera* genomes and aphid genomes varied significantly between the 4^th^ instars of the clones tested. In 4^th^ instar aphids, *Buchnera* genome copy number explained 62.9% of the variation in titer (p < 0.0001, ANOVA) while the aphid genome accounted for 18.5% (p < 0.0001, ANOVA) based on measuring effects of each variable independently.

### 3.2. Titer in F_1_ Clones

**Figure 3:**
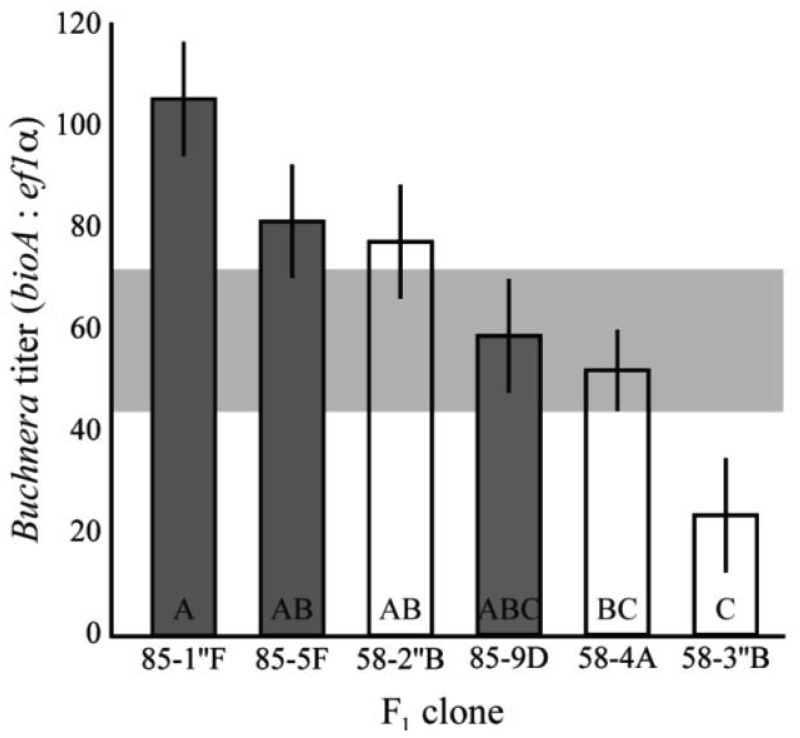
Symbiont titer in 1^st^ instar F_1_ offspring clones from a reciprocal cross of 8-10-1 and 5A. Clones varied significantly (F_6,41_ = 2.9, p = 0.0004, ANCOVA), though there was no significant effect of matriline (p = 0.83, t-test). Open bars are 5A matriline, closed bars are 8-10-1 matriline. Matriline indicates same *Buchnera* genotype. Shaded area represents the range of titer observed in clones originating from field-collected asexual females. Bars with different letters are significantly different (p < 0.05, Tukey's HSD). Error bars are ± SE.

To determine the pattern of heritability in titer, clones 5A and 8-10-1 were reciprocally mated to produce a panel of full-sib clones with distinct genotypes. The *Buchnera* titer of 1^st^ instar aphids were significantly different among F_1_ clones (F_6,93_ = 2.9, p = 0.0004, ANCOVA, Figure 3). The clones also had significantly higher and lower average titers than those observed in the lab clones–those established from field collected asexual females–or their parental clones (F_1,15_ = 8.48, p = 0.0042, ANCOVA). Titers ranged from 23.4 *Buchnera* genomes per aphid genome in clone 58-3″B and 105.6 in clone 85-1″F. These values were significantly lower and higher than those observed in the parental clones (p < 0.05, Tukey's HSD).

As *Buchnera* are maternally transmitted, aphids from the two matrilines have different *Buchnera* genotypes. There was no significant difference observed in the *Buchnera* titer of F_1_ clones from the two matrilines, suggesting that differences between the genomes of *Buchnera* from 5A and 8-10-1 are not responsible for the differences in symbiont titer between these two clones (F_1,93_ = 0.046, p = 0.83, ANCOVA).

### 3.3. Bacteriocyte Quantification and *Buchnera* per Bacteriocyte

**Figure 4:**
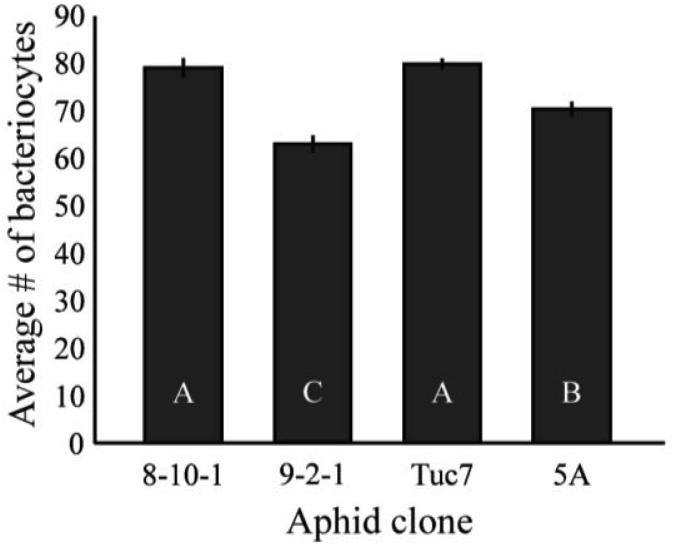
Average number of bacteriocytes in 4^th^ instar *A. pisum* clones. Bacteriocyte counts were significantly different between clones tested (F_3,172_ = 21.3, p < 0.0001, LSM). Bars with different letters are significantly different (p < 0.05, Tukey's HSD). Error bars are ± SE.

Figure 4 shows the average number of bacteriocytes per aphid at the 4^th^ instar. Bacteriocyte counts varied significantly between clones tested (F_3,172_ = 21.3, p < 0.0001, ANOVA). These data are mostly consistent with the *Buchnera* titer of 4^th^ instar aphids (Figure 2), with Tuc7 and 8-10-1 having similar numbers of bacteriocytes per aphid while 9-2-1 and 5A have significantly fewer (p < 0.05, Tukey's HSD). Clone 9-2-1 has fewer bacteriocytes than clone 5A (p < 0.05, Tukey's HSD), though these clones do not differ in the titer of *Buchnera* at the 4^th^ instar (Figure 2). While variation in *Buchnera* titer in 1^st^ instars is not consistently reflected in measures of number of bacteriocytes or the number of *Buchnera* per bacteriocyte, the symbiont titer of 4^th^ instar aphids recapitulates the patterns observed in the number of bacteriocytes in the clones tested, suggesting that control of bacteriocyte number is one mechanism by which the symbiont populations within an individual is regulated by the host.

**Figure 5:**
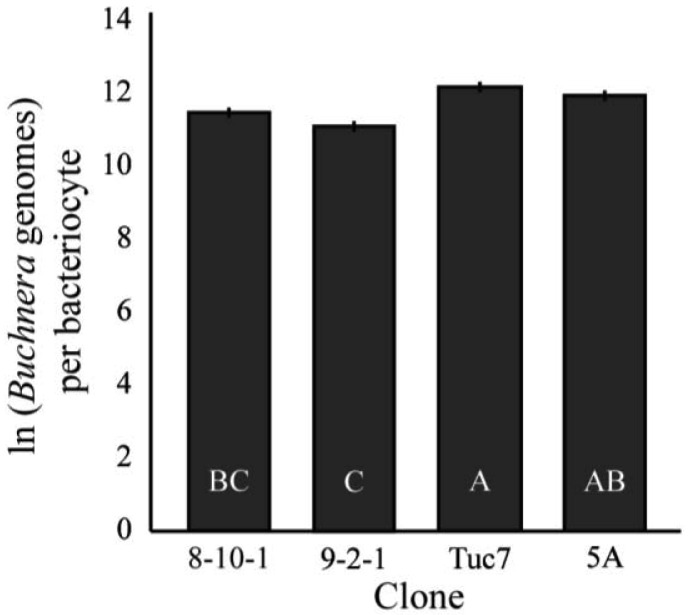
Comparison of natural log of *Buchnera* genomes per aphid bacteriocyte. (F_3,3_ = 11.68, p < 0.0001, MANOVA). Bars with different letters are significantly different (p < 0.05, Tukey's HSD). Error bars are ± SE.

The number of *Buchnera* genomes per bacteriocyte was significantly different among the clones examined (F_3,94_ = 10.24, p < 0.0001, MANOVA, Figure 5). 9-2-1 had the fewest *Buchnera* per bacteriocyte, significantly fewer than all the clones except 8-10-1 (p < 0.05, Tukey's HSD). Tuc7 and 5A had an equivalent number of *Buchnera* per bacteriocyte, while 8-10-1 had fewer than Tuc7, but the difference between 5A and 8-10-1 was not significant.

**Figure S1:**
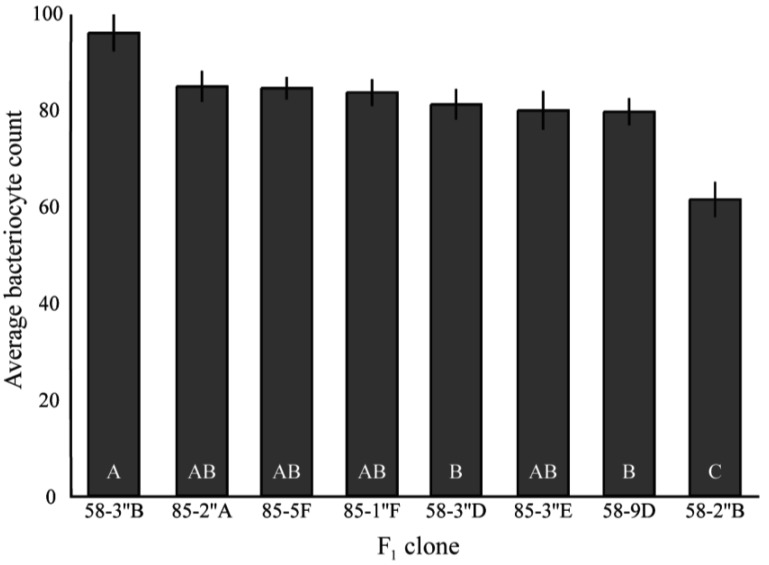
Average number of bacteriocytes from F_1_ *A. pisum* Clones labeled 58 are from the clone 5A matriline, while clones labeled 85 are from the 8-10-1 matriline. Clones exhibited significant differences F_5,106_ = 8.00, p < 0.0001, ANOVA. Bars with different letters are significantly different (p < 0.05, Tukey's HSD).

Together, results for bacteriocyte numbers are consistent with the differences in total *Buchnera* titers in three of the four clones examined. 5A and 8-10-1 exhibited the largest difference in 1^st^ instar *Buchnera* titer, and 8-10-1 had significantly more bacteriocytes than 5A. In these clones, there was no significant difference between the numbers of *Buchnera* genomes per bacteriocyte, suggesting that the number of bacteriocytes may be responsible for the difference in *Buchnera* titer between the clones. Tuc7 had more bacteriocytes than 5A and more *Buchnera* per bacteriocyte than 8-10-1, though it had intermediate 1^st^ instar titer compared to these clones. While there was a strong correlation between bacteriocyte numbers and *Buchnera* titer for the four clones tested (R^2^ = 0.62), the relationship was not significant (F_1,4_ = 3.39, p = 0.2, ANCOVA) though a lack of power likely contributed to this. Significant variation in bacteriocyte numbers was also seen in the F_1_ clones (F_5,106_ = 8.00, p < 0.0001, ANOVA, Figure S1), though no comparison could be made between the *Buchnera* titer and bacteriocyte number in these clones, as they were measured at 1^st^ and 4^th^ instars, respectively.

### 3.4. *Buchnera* Titer and Amino Acid Requirements of Clones

**Figure S2:**
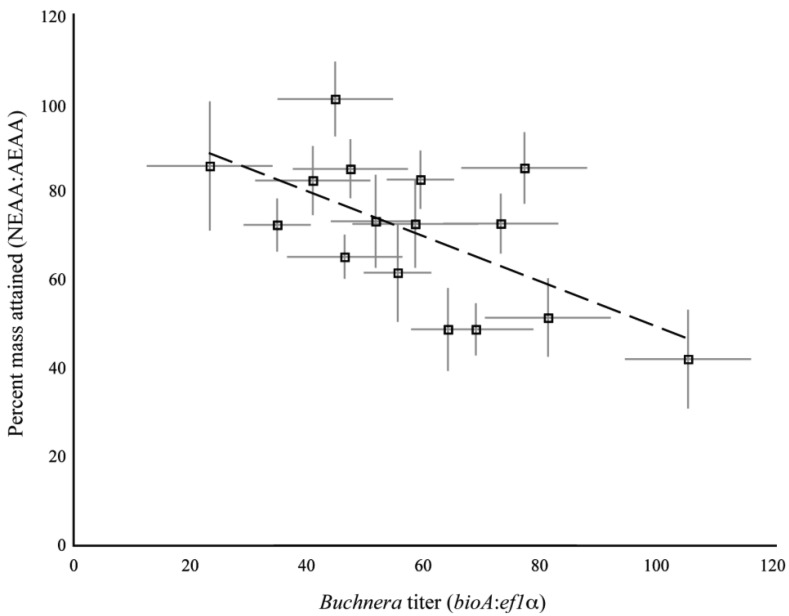
Correlation of Buchnera titer and amino acid requirements for all clones. Each point represents the mean of a clone, error bars are +/- SE. The relationship was significant, R^2^ = 0.38, F_1,14_ = 8.71, p = 0.01, ANCOVA.

The clones used in the present study were previously assayed for their dietary requirements of essential amino acids [9]. In that study, we measured the mass of aphids reared on artificial diets with and without essential amino acids. We used the ratio of aphid mass on the diet without essential amino acids to the mass of aphids reared on diet with all the essential amino acids as a measure of the dietary requirement for essential amino acids for each clone. As different individuals were measured for the previous study and the current study, we compared the mean amino acid requirement of a clone against the mean 1^st^ instar *Buchnera* titer as measured in the current study. We found a significant, positive association between amino acid requirements and *Buchnera* titer for the set of clones tested (R^2^ = 0.38, F_1,14_ = 8.71, p = 0.01, ANCOVA, Figure S2).

## 4. Conclusions

Control of symbiont titer varies among clonal lines of pea aphid. The titer of *Buchnera*, defined as number of *Buchnera* genomes relative to aphid genomes, can also vary with age of the aphid, increasing significantly between the 1^st^ and 4^th^ instar in two of the four clones tested. Part of the variation in *Buchnera* titer may be due to differences in the number of bacteriocytes between clones. There is significant variation in the number of *Buchnera* genomes per bacteriocyte, but this was not paralleled by variation in overall *Buchnera* titer. In both the current study and previous research, the number of bacteriocytes has been shown to vary between *A. pisum* clones [19].

A moderate positive association was found between the essential amino acid requirements of the clones and the titer of *Buchnera*. Previous work has shown that deleterious mutations accumulate in the genome of *Buchnera* [20], including the amino acid biosynthesis genes of *Buchnera* [9], though recent studies have revealed that some classes of deleterious mutations can be overcome by translational slippage [21,22]. It is possible that the increased number of *Buchnera* genomes in clones with dietary amino acid requirements is a compensatory change that increases the number of functional transcripts produced from the inactivated gene.

The mechanistic basis for variation in symbiont titer is unclear, though several mechanisms may contribute. Bacteriocyte development involves the interactions of multiple genes during development, and differences in the expression of these genes or timing of expression between clones could impact titer [18]. Additionally, host lysozyme-like genes expressed in the bacteriocytes have been shown to degrade *Buchnera* and bacteriocytes in post-reproductive aphids [23]. While the current study focused on pre-reproductive aphids, it is possible that variation in expression of lysozyme genes between clones contributes to differences in symbiont titer. Recent theoretical work has suggested that the aphid can manipulate the metabolism of *Buchnera* by regulating the supply of precursor metabolites [24], and variation in the supply of metabolites may have a profound impact on *Buchnera's* replication and division. Variation in the DNA sequence of the genes involved in these processes, their expression, or their interaction could all impact symbiont titer.

The lack of a maternal effect in the titer of F_1_ clones indicates that *Buchnera* is not primarily responsible for the observed wide variation in titer among aphid clones. This finding is consistent with previous work indicating that *Buchnera* lacks basic regulatory mechanisms, as well as the absence of many genes involved in cell cycle control within the symbiont genomes. However, the differences between the *Buchnera* genomes of the parental clones are minimal, with a total of 9 point mutations [6], and it is possible that additional genomic differences between *Buchnera* strains could further affect titer. Nonetheless, we observed wide variation in titer attributable to host genotype.

Selection appears to favor clones with an intermediate titer within a 2-fold range as evidenced by the titers observed in field-collected clones. These observations, along with the 4-fold variation in F_1_ symbiont titer, indicates that extremely high or low symbiont levels are likely maladaptive. The extensive variation seen among F_1_ clones, which displayed titers much higher and much lower than either parent, suggests epistatic or dominance effects on titer, or the occurrence of high levels of heterozygosity in the parental genotypes at loci affecting titer. Because only six F_1_ clones were tested, from a single cross, we cannot estimate heritability of this trait. During clonal reproduction, selection may remove clones with extreme titers from the population, resulting in the intermediate titer observed in field-collected clones. However, the phenotype appears non-heritable across phases of sexual reproduction, resulting in reappearance of clones with extreme titers each spring, following the annual sexual generation of most populations. It is remarkable that symbiont titer, which is likely an important factor in aphid fitness, exhibits such variation after sexual reproduction.

## Figures and Tables

**Table S1:** Aphid collection information.

**Clone**	**Collection Date**	**Collection Location**	**Collected by**	**Host Plant**
8.10.1	2001	Cayuga Co., NY	J. Russell	*Medicago sativa*
9.2.1	2001	Cayuga Co., NY	J. Russell	*Medicago sativa*
Tuc7	2007	Tucson, AZ	N. Moran	*Medicago sativa*
5A	1999	Madison, WI	N. Moran	*Medicago lupulina*
Alm	2008	Whitman Co., WA	S. Eigenbrode	*Pisum sativum*
File	2008	Whitman Co., WA	S. Eigenbrode	*Pisum sativum*
Cag	2007	Walnut Creek, CA	N. Moran	*Medicago lupulina*
7A	2001	Cayuga Co., NY	J. Russell	*Medicago sativa*

**Table S2:** Summary statistics of experiments.

**Factor**	**F**	**p**
Titer of field-collected clones		
*bioA* copy #^1^	F_9, 95_ = 5.26	< 0.0001
*ef1α* copy #^1^	F_9, 95_ = 5.80	< 0.0001
Clone^2^	F_9, 95_ = 3.21	0.0019
Titer of F_1_ aphid clones		
Clone^2^	F_6, 93_ = 2.90	0.0004
[Matriline] Clone^2^	F_1, 93_ = 0.046	0.83
*bioA* copy #^1^	F_18, 93_ = 3.13	0.0002
*ef1α* copy #^1^	F_18, 93_ = 2.13	0.0099
Titer of 4th instar aphids		
Clone^2^	F_3, 97_ = 23.12	< 0.0001
Instar^2^	F_1, 97_ = 24.19	< 0.0001
Clone × Instar^2^	F_7, 97_ = 10.37	< 0.0001
Bacteriocyte counts		
Field collected clones^3^	F_3,172_ = 21.31	<0.0001
F_1_ clones^3^	F_5,106_ = 8.00	<0.0001
Buchnera per bacteriocyte		
Clone^1^	F_3, 94_ = 10.24	< 0.0001
[Bacteriocyte] Clone^1^	F_16, 94_ = 0.498	0.942

1 ANOVA,

2 ANCOVA,

3 MANOVA
